# Activity of cabozantinib in radioresistant brain metastases from renal cell carcinoma: two case reports

**DOI:** 10.1186/s13256-018-1875-9

**Published:** 2018-11-25

**Authors:** Sylvie Négrier, Guillaume Moriceau, Valéry Attignon, Véronique Haddad, Daniel Pissaloux, Nicole Guerin, Christian Carrie

**Affiliations:** 1 Univ Lyon, Université Claude Bernard Lyon 1, Lyon, France; 2 Department of Medical Oncology, Centre Léon Bérard, Lyon, France; 3 Department of Translational Research and Innovation, Centre Léon Bérard, Lyon, France; 4 Department of Bio-Pathology, Centre Léon Bérard, Lyon, France; 5 Department of Radiology, Centre Léon Bérard, Lyon, France; 6 Department of Radiation Therapy, Centre Léon Bérard, Lyon, France

**Keywords:** Renal cell carcinoma, Brain metastases, Cabozantinib, Tyrosine kinase inhibitor, VEGFr inhibitors

## Abstract

**Background:**

Renal cell carcinoma represents 3–5% of adult malignant tumors. Metastases are found in 30–40% of patients and brain metastases occurred in more than 10% of them. Despite significant progress in medical treatment, patients with brain metastases still have a limited survival. Cabozantinib, a tyrosine kinase inhibitor directed against vascular endothelial growth factor receptors, was recently registered for the treatment of metastatic renal cell carcinoma. Almost no data are, however, available on patients with brain metastases.

**Case presentation:**

Case 1 is a 51-year-old man of North African origin; Case 2 is a 55-year-old European man. Case 1 and Case 2 had metastases of renal carcinoma at initial diagnosis and were treated with vascular endothelial growth factor receptors tyrosine kinase inhibitors. Case 1 had clear cell renal carcinoma and underwent nephrectomy; he then received several lines of tyrosine kinase inhibitor directed against vascular endothelial growth factor receptors and the mTor complex. During the second treatment a brain metastasis was diagnosed and treated with radiosurgery with rapid efficacy. Two years later he received nivolumab, an antibody directed against the programmed death-1 and programmed death-ligand 1 complex, but disease progression was observed with the reappearance of the brain metastasis together with neurologic symptoms. Cabozantinib was administered and induced a rapid clinical improvement as well as tumor regression in all sites including his brain. Sequencing of his tumor evidenced a mutation of the *MET* gene. Case 2 had a papillary renal carcinoma with brain metastases at time of diagnosis. After radiation of the brain tumors, a vascular endothelial growth factor receptor tyrosine kinase inhibitor was administered for 3 years. The disease was under control in all sites except in his brain; several new brain metastases requiring new radiation treatments developed. The disease finally progressed at all metastatic sites including his brain and he had several neurological symptoms. Cabozantinib was administered and rapidly induced a clinical improvement; a further computed tomography scan and brain magnetic resonance imaging showed significant tumor regressions. No *MET* gene mutation or amplification was observed in the tumor analysis.

**Conclusions:**

These case reports indicate that cabozantinib was able, first, to reach brain tumors and second, to induce significant regressions in renal carcinoma brain metastases that were resistant to radiation as well as to previous systemic vascular endothelial growth factor receptor tyrosine kinase inhibitors.

## Background

Renal carcinoma represents 3–5% of the incidence of adult malign tumors. The more common histologic subtype is clear cell carcinoma and it accounts for more than 75% of cases; several minor subtypes are diagnosed among the remaining 25% of cases. Papillary carcinoma, type I or II, is the most frequent of these minor subtypes and accounts for 10% of all cases. Surgery with partial or radical nephrectomies cures most patients and disease-specific survival at 5 years is between 70 and 80% [1, 2]. Metastases often occur in the 2 to 5 years following surgery but are uncommon at initial diagnosis. Metastases are frequently located in lungs and lymph nodes, but also in bones and liver; brain metastases are unfrequently observed with an estimated incidence of 10% [3, 4]. Brain metastases are generally associated with a limited survival time despite local specific treatments with neurosurgery or radiation therapy [3, 5]. Significant progress in the treatment of metastatic renal cell carcinoma (mRCC) was achieved in the past decade; however, patients die after a survival period varying from 1 to 3–4 years depending on the prognosis factors [1]. Due to specific gene alterations, the vascular endothelial growth factor (VEGF) pathway is a major driver of clear cell renal carcinoma development, which is the most frequent histologic subtype, and, as a consequence, VEGF or VEGF receptor (VEGFr) inhibitors are key in the treatment of these patients [1]. Other genes, especially *MET*, involved in the carcinogenesis of a number of tumors, were also found to be determinants for tumor progression and resistance to treatments. MET was first identified as a major driver for the development of papillary renal carcinoma, but was also shown to be involved in resistant clear cell carcinomas [6–8]. Different MET or dual MET and VEGF-targeted therapies were recently investigated through clinical trials [9, 10]. One of the most recently registered targeted therapies for the treatment of mRCC, cabozantinib, is a tyrosine kinase inhibitor (TKI) directed against VEGFrs, but also against other different genes: c-Met, RET, and AXL [11].

We describe two cases of mRCC who developed resistant brain metastases despite several systemic treatments as well as stereotaxic radiation. Cabozantinib induced significant tumor reductions including the life-threatening brain metastases.

## Case presentation

**Fig. 1:**
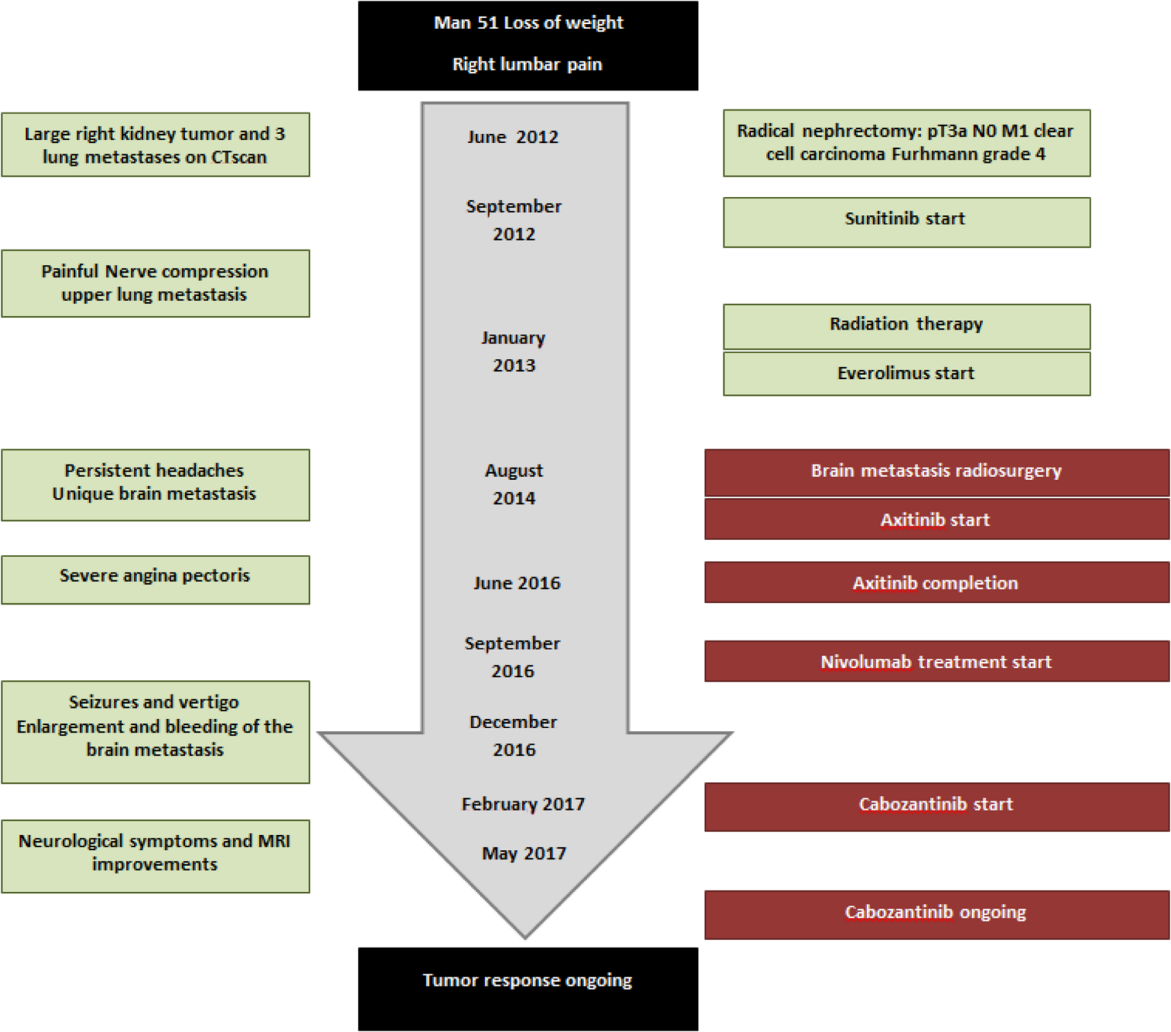
Case 1 timeline. *CT* computed tomography, *MRI* magnetic resonance imaging

**Fig. 2:**
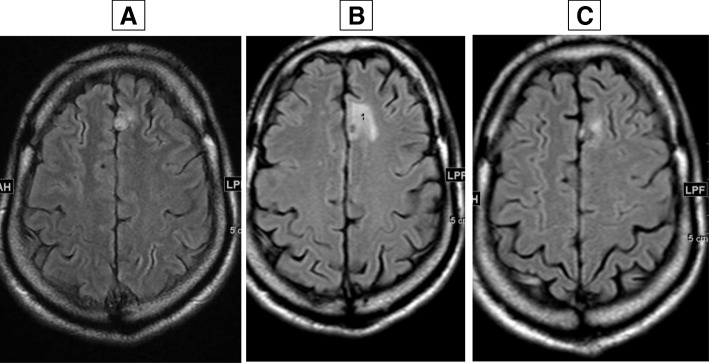
Brain magnetic resonance imaging of Case 1. **a** July 2016, **b** December 2016, **c** May 2017 4 months under cabozantinib

Case 1 is a 51-year-old man of North African origin with a history of hypertension who had been diagnosed as having a right kidney tumor associated with one bulky pleural metastasis and some smaller metastatic lesions of the lung; Case 1 is summarized in Fig. 1. No bone or brain metastases were observed at initial work up; he was classified in the poor risk group according to the International Metastatic RCC Database Consortium (IMDC) [12]. A radical nephrectomy was performed in July 2012. A pathological report indicated a renal cell carcinoma (RCC) of 16 cm with a clear cell component and some degree of a more aggressive cellular component, giving a Fuhrman grade of 4, pT3a pN0 M1 according to the Union for International Cancer Control (UICC) classification. Sunitinib, 50 mg/day, then reduced to 37.5 mg due to side effects, was administered during 6 months. Because of the painful progression of the pleural metastasis in the upper part of his left lung, radiation therapy was delivered to this tumor. Systemic treatment was further modified for the approved second-line treatment everolimus. This latter treatment induced a significant tumor response in most metastatic sites for 15 months before re-progression. In March 2014, our patient complained of persistent headaches and brain magnetic resonance imaging (MRI) identified a single right frontal metastasis. Stereotactic radiotherapy was performed and a treatment with axitinib, a second-line TKI directed against VEGFrs, was started. Axitinib induced significant tumor shrinkage in the pleural and lung metastases; the brain metastasis was much improved because a brain MRI was considered almost normal. Axitinib was maintained for 18 months, but had to be completed because of a severe episode of angina pectoris. A coronary stent that required dual anti-platelet therapy for 6 months was indicated. Due to an increased hemorrhagic risk with this treatment together with a VEGFr inhibitor, axitinib was not resumed; nivolumab, a programmed death-1 (PD1) directed antibody recently approved for mRCC treatment, was administered as part of a compassionate program. Our patient was admitted with seizures and vertigo 4 months after immunotherapy initiation. A brain MRI evidenced the enlargement, with some hemorrhagic traits, of the previously treated metastasis. Further to therapeutic control of his neurological symptoms, a thoracic and abdominal computed tomography (CT) scan showed the progression of the disease at all metastatic sites. He then entered a genetic profiling experimental program (NCT 01774409) and sequencing of the primary tumor was performed. Due to the long duration of this analysis, cabozantinib that had been recently made available was administered after our patient gave consent. All neurological symptoms disappeared and his performance status (PS) score improved to 0 after 2 weeks of treatment with cabozantinib. A brain MRI and thoracoabdominal CT scan at 8 weeks indicated a significant shrinkage of the metastatic lesions including the brain metastasis (Fig. 2). Treatment was rather well tolerated but had to be reduced to 40 mg per day instead of 60 mg, due to fatigue, stomatitis, and loss of weight. Cabozantinib is ongoing with a reduced dose of 40 mg/day with a maintained efficacy over 8 months. Meanwhile, results of the tumor sequencing indicated the expression of mutation of the *MET* gene.

**Fig. 3:**
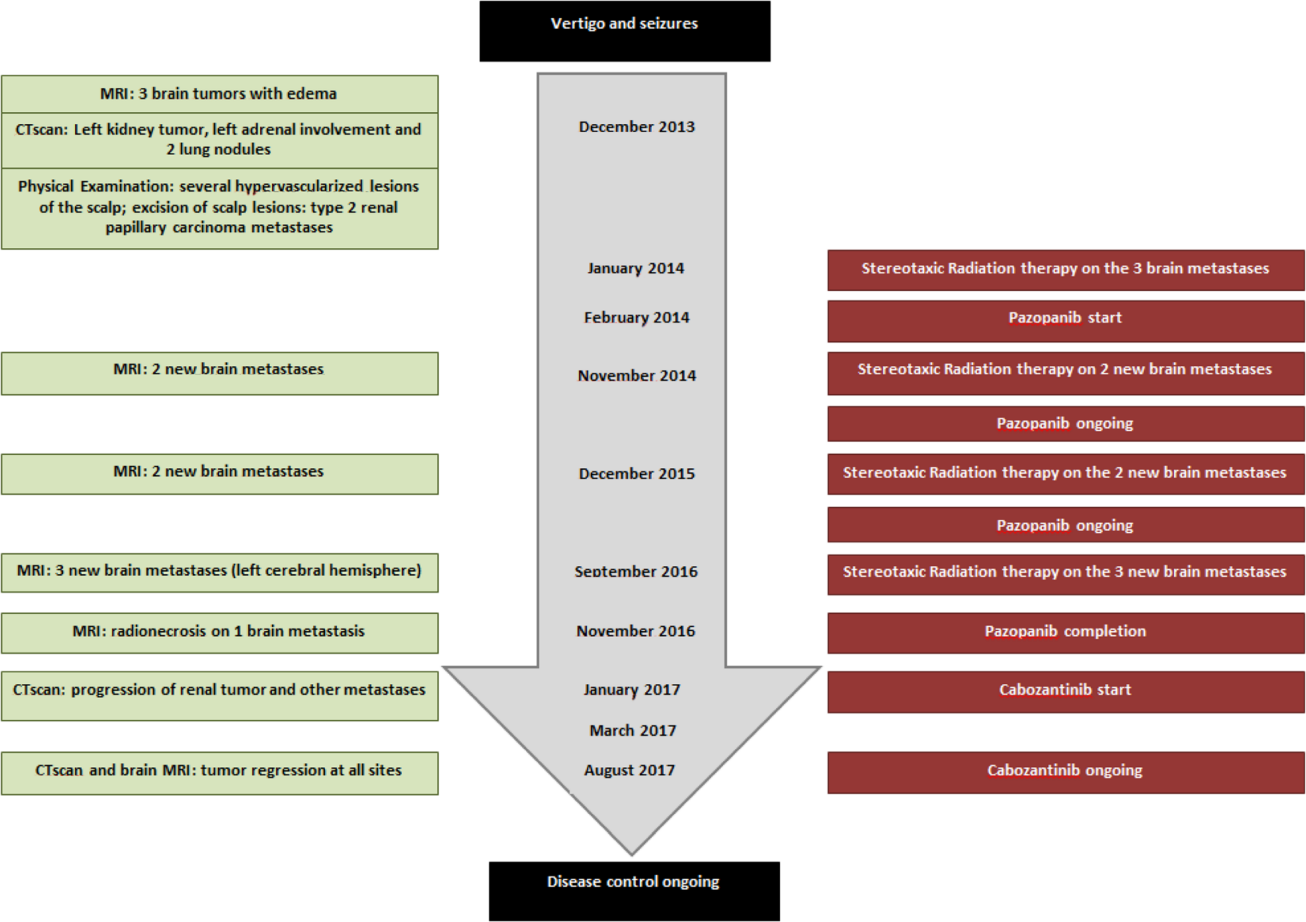
Case 2 timeline. *CT* computed tomography, *MRI* magnetic resonance imaging

**Fig. 4:**
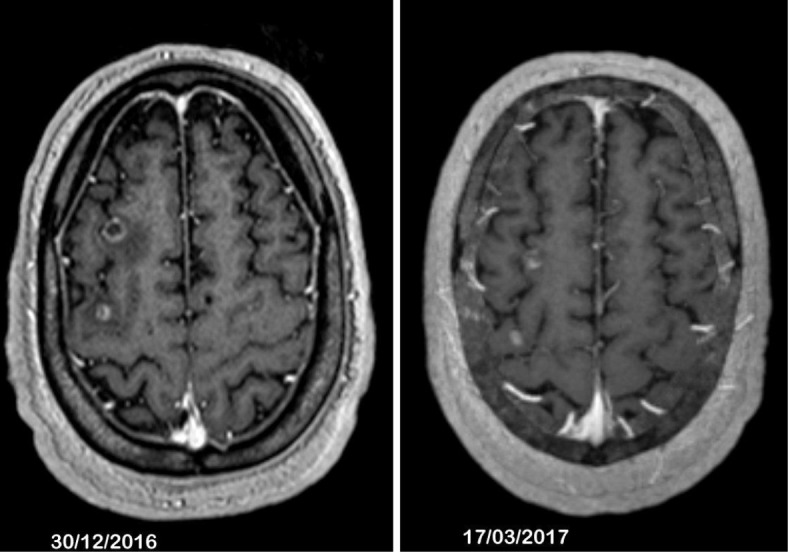
Brain magnetic resonance imaging of Case 2, effect of cabozantinib after 2.5 months

Case 2 is a 55-year-old European man with a history of hypertension who presented to the emergency room with seizures in December 2013; Case 2 is summarized in Fig. 3. A brain CT scan and further MRI showed three tumors surrounded by cerebral edema. A left kidney tumor and two lung nodules were identified by CT scan and, finally, clinical examination found some hypervascularized lesions of his scalp. The cutaneous tumors were surgically removed and the pathological report identified metastases of a type 2 papillary renal tumor. This patient was classified in the favorable risk group according to the International Metastatic RCC Database Consortium (IMDC) [12]. Brain metastases were all treated by stereotaxic radiation. Pazopanib another TKI directed to VEGFr was initiated at 800 mg/day. This treatment induced a partial response in lung metastases and in the primary renal tumor; the three brain metastases were also reduced. The disease remained stable for 2.5 years under pazopanib, except in his brain. In fact, two new brain metastases appeared 12 months later and three others after 24 months. Stereotaxic radiation was performed on each new brain tumor and pazopanib at 800 mg per day was resumed. Some neurological symptoms appeared with several transient episodes of aphasia together with some degree of mental confusion, 4 months after the last radiation treatment. Pazopanib treatment was completed and brain MRI indicated a radionecrosis with surrounding cerebral edema in one of the recently irradiated brain metastases. Two months after pazopanib completion, a CT scan showed significant progression in all other metastatic sites including previously irradiated brain metastases. Cabozantinib was started after our patient gave consent. Neurological symptoms rapidly resolved and a brain MRI at 2.5 months evidenced tumor regression of the different brain metastases (Fig. 4). Cabozantinib was ongoing for 6 months but had to be reduced to 40 mg/day due to grade 3 diarrhea. Sequencing was performed on the metastatic tumor sample but no *MET* mutation was identified and no *MET* gene amplification was observed.

## Discussion

To the best of our knowledge, this is one the two first reports of efficacy of cabozantinib in brain metastases of RCC. Cabozantinib is a TKI directed against VEGFrs as well as MET, RET and AXL gene expression [11, 13]. Cabozantinib was approved by both the US Food and Drug Administration and the European Medicines Agency in 2016 for the treatment of mRCC after failure of previous treatments with angiogenesis inhibitors. In fact, cabozantinib was shown to improve progression-free survival (PFS) and overall survival over everolimus in patients with advanced or mRCC [11, 14]. More recently, PFS with cabozantinib was found superior to sunitinib in patients with non-pretreated mRCC in a randomized phase 2 trial [15]. None of these trials enrolled patients with active brain metastases; as a consequence, the potential efficacy of cabozantinib is unknown at this specific tumor site. After a review of the literature, we found a case report on the regression of brain metastases in a patient with RCC and one non-small cell lung carcinoma (NSCLC) under cabozantinib [16, 17]. These cases demonstrated the potential activity of cabozantinib on the brain, which is an unfavorable site in all patients with metastases. In addition, our two cases demonstrate the potential efficacy of cabozantinib in radioresistant brain metastases of RCC. Some retrospective analyses reported some tumor regression in brain metastases under other VEGFr inhibitors, mostly sunitinib, but efficacy appeared limited when investigated in a specific trial [3, 18, 19]. A hypothesis of a correlation between the effect of cabozantinib on resistant brain metastases and MET inhibition can be raised. In our first case, cMet was found mutated and the second case has a papillary carcinoma which is known to commonly have *MET* gene alterations, although no mutation in this gene was found in the tumor of this patient [6, 20]. Notably, a *MET* mutation was present in the case of NSCLC with brain metastases successfully treated by cabozantinib [17]. Recently, some results indicated more frequent *MET* gene mutations or overexpression in brain metastases of patients with mRCC compared to primary tumor or other metastatic sites [21, 22].

## Conclusions

These case reports indicate that orally administered cabozantinib is able to reach brain tumors and to induce regressions in metastases from RCC that were resistant to radiation and previous angiogenic TKI. A prospective trial with cabozantinib in patients with mRCC and brain metastases is required, as well as further investigations on the role of the *MET* gene in these patients.

## Abbreviations

CT: Computed tomography
IMDC: International Metastatic RCC Database Consortium
mRCC: Metastatic renal cell carcinoma
MRI: Magnetic resonance imaging
NSCLC: Non-small cell lung carcinoma
PD1: Programmed death-1
PFS: Progression-free survival
PS: Performance status
RCC: Renal cell carcinoma
TKI: Tyrosine kinase inhibitor
UICC: Union for International Cancer Control
VEGF: Vascular endothelial growth factor
VEGFr: Vascular endothelial growth factor receptor

## References

[1] Choueiri TK, Motzer RJ. Systemic Therapy for Metastatic Renal-Cell Carcinoma. N Engl J Med. 2017;376:354–66. 10.1056/NEJMra1601333 28121507

[2] Znaor A, Lortet-Tieulent J, Laversanne M, Jemal A, Bray F. International variations and trends in renal cell carcinoma incidence and mortality. Eur Urol. 2015;67(3):519–30. 10.1016/j.eururo.2014.10.002 25449206

[3] Gore ME, Hariharan S, Porta C, Bracarda S, Hawkins R, Bjarnason GA, Oudard S, Lee SH, Carteni G, Nieto A, Yuan J, Szczylik C. Sunitinib in metastatic renal cell carcinoma patients with brain metastases. Cancer. 2011;117(3):501–9. 10.1002/cncr.25452 20862748

[4] Cagney DN, Martin AM, Catalano PJ, Redig AJ, Lin NU, Lee EQ, Wen PY, Dunn IF, Bi WL, Weiss SE, Haas-Kogan DA, Alexander BM, Aizer AA. Incidence and prognosis of patients with brain metastases at diagnosis of systemic malignancy: A population-based study. Neuro-Oncology. 2017;19:1511–21. 10.1093/neuonc/nox077. PMC5737512 28444227

[5] Chandrasekar T, Klaassen Z, Goldberg H, Kulkarni GS, Hamilton RJ, Fleshner NE. Metastatic renal cell carcinoma: Patterns and predictors of metastases-A contemporary population-based series. Urol Oncol. 2017;35:661.e7–661.e14. 10.1016/j.urolonc.2017.06.060. 28728748

[6] Linehan, *et al.* Comprehensive Molecular Characterization of Papillary Renal-Cell Carcinoma. N Engl J Med. 2016;374(2):135–45. 10.1056/NEJMoa1505917 PMC4775252 26536169

[7] Shojaei F, Lee JH, Simmons BH, Wong A, Esparza CO, Plumlee PA, Feng J, Stewart AE, Hu-Lowe DD, Christensen JG. HGF/c-Met acts as an alternative angiogenic pathway in sunitinib-resistant tumors. Cancer Res. 2010;70(24):10090–100. 10.1158/0008-5472.CAN-10-0489 20952508

[8] Gibney GT, Aziz SA, Camp RL, Conrad P, Schwartz BE, Chen CR, Kelly WK, Kluger HM. c-Met is a prognostic marker and potential therapeutic target in clear cell renal cell carcinoma. Ann Oncol. 2013;24(2):343–9. 10.1093/annonc/mds463 PMC3551486 23022995

[9] Choueiri TK, Vaishampayan U, Rosenberg JE, Logan TF, Harzstark AL, Bukowski RM, Rini BI, Srinivas S, Stein MN, Adams LM, Ottesen LH, Laubscher KH, Sherman L, McDermott DF, Haas NB, Flaherty KT, Ross R, Eisenberg P, Meltzer PS, Merino MJ, Bottaro DP, Linehan WM, Srinivasan R. Phase II and biomarker study of the dual MET/VEGFR2 inhibitor foretinib in patients with papillary renal cell carcinoma. J Clin Oncol. 2013;31(2):181–6. 10.1200/JCO.2012.43.3383 PMC3532390 23213094

[10] Choueiri JCO, Choueiri TK, Plimack E, Arkenau HT, Jonasch E, DYC H, Powles T, Frigault MM, Clark EA, Handzel AA, Gardner H, Morgan S, Albiges L, Pal SK. Biomarker-Based Phase II Trial of Savolitinib in Patients With Advanced Papillary Renal Cell Cancer. J Clin Oncol. 2017;35(26):2993–3001. 201710.1200/JCO.2017.72.2967 10.1200/JCO.2017.72.2967 28644771

[11] Choueiri TK, Escudier B, Powles T, Mainwaring PN, Rini BI, Donskov F, Hammers H, Hutson TE, Lee JL, Peltola K, Roth BJ, Bjarnason GA, Géczi L, Keam B, Maroto P, Heng DY, Schmidinger M, Kantoff PW, Borgman-Hagey A, Hessel C, Scheffold C, Schwab GM, Tannir NM, Motzer RJ, METEOR Investigators. Cabozantinib versus Everolimus in Advanced Renal-Cell Carcinoma. N Engl J Med. 2015;373(19):1814–23. 10.1056/NEJMoa1510016 PMC5024539 26406150

[12] Heng DY, Xie W, Regan MM, Warren MA, Golshayan AR, Sahi C, Eigl BJ, Ruether JD, Cheng T, North S, Venner P, Knox JJ, Chi KN, Kollmannsberger C, McDermott DF, Oh WK, Atkins MB, Bukowski RM, Rini BI, Choueiri TK. Prognostic factors for overall survival in patients with metastatic renal cell carcinoma treated with vascular endothelial growth factor-targeted agents: results from a large, multicenter study. J Clin Oncol. 2009;27(34):5794–9. 10.1200/JCO.2008.21.4809 19826129

[13] Yakes FM, Chen J, Tan J, Yamaguchi K, Shi Y, Yu P, Qian F, Chu F, Bentzien F, Cancilla B, Orf J, You A, Laird AD, Engst S, Lee L, Lesch J, Chou YC, Joly AH. Cabozantinib (XL184), a novel MET and VEGFR2 inhibitor, simultaneously suppresses metastasis, angiogenesis, and tumor growth. Mol Cancer Ther. 2011;10(12):2298–308. 10.1158/1535-7163.MCT-11-0264 21926191

[14] Choueiri TK, Escudier B, Powles T, Tannir NM, Mainwaring PN, Rini BI, Hammers HJ, Donskov F, Roth BJ, Peltola K, Lee JL, Heng DYC, Schmidinger M, Agarwal N, Sternberg CN, McDermott DF, Aftab DT, Hessel C, Scheffold C, Schwab G, Hutson TE, Pal S, Motzer RJ, METEOR investigators. Cabozantinib versus everolimus in advanced renal cell carcinoma (METEOR): final results from a randomised, open-label phase 3 trial. Lancet Oncol. 2016;17(7):917–27. 10.1016/S1470-2045(16)30107-3 27279544

[15] Choueiri TK, Halabi S, Sanford BL, Hahn O, Michaelson MD, Walsh MK, Feldman DR, Olencki T, Picus J, Small EJ, Dakhil S, George DJ, Morris MJ. Cabozantinib Versus Sunitinib As Initial Targeted Therapy for Patients With Metastatic Renal Cell Carcinoma of Poor or Intermediate Risk: The Alliance A031203 CABOSUN Trial. J Clin Oncol. 2017;35(6):591–7. 10.1200/JCO.2016.70.7398 PMC5455807 28199818

[16] Ciccarese C, Iacovelli R, Mosillo C, Tortora G. Exceptional response to cabozantinib of rapidly evolving brain metastases of renal cell carcinoma : a case report and review of the literature. Clin Genitourin Cancer. 2018; 10.1016/J.clcg.2018.06.005. 30005936

[17] Klempner SJ, Borghei A, Hakimian B, Ali SM, Ou SI. Intracranial Activity of Cabozantinib in MET Exon 14-Positive NSCLC with Brain Metastases. J Thorac Oncol. 2017;12(1):152–6. 10.1016/j.jtho.2016.09.127 27693535

[18] Bianchi M, Sun M, Jeldres C, Shariat SF, Trinh QD, Briganti A, Tian Z, Schmitges J, Graefen M, Perrotte P, Menon M, Montorsi F, Karakiewicz PI. Distribution of metastatic sites in renal cell carcinoma: a population-based analysis. Ann Oncol. 2012;23(4):973–80. 10.1093/annonc/mdr362 21890909

[19] Chevreau C, Ravaud A, Escudier B, Amela E, Delva R, Rolland F, Tosi D, Oudard S, Blanc E, Ferlay C, Négrier S, French Group on Renal Cancer. A phase II trial of sunitinib in patients with renal cell cancer and untreated brain metastases. Clin Genitourin Cancer. 2014;12(1):50-4. 10.1016/j.clgc.2013.09.008 24268852

[20] Albiges L, Guegan J, Le Formal A, Verkarre V, Rioux-Leclercq N, Sibony M, Bernhard JC, Camparo P, Merabet Z, Molinie V, Allory Y, Orear C, Couvé S, Gad S, Patard JJ, Escudier B. MET is a potential target across all papillary renal cell carcinomas: result from a large molecular study of pRCC with CGH array and matching gene expression array. Clin Cancer Res. 2014;20(13):3411–21. 10.1158/1078-0432.CCR-13-2173 24658158

[21] Schiefer AI, Mesteri I, Berghoff AS, Haitel A, Schmidinger M, Preusser M, Birner P. Evaluation of tyrosine kinase receptors in brain metastases of clear cell renal cell carcinoma reveals cMet as a negative prognostic factor. Histopathology. 2015;67(6):799–805. 10.1111/his.12709 25847631

[22] Derosa L, *et al.* Inter and intra-tumor heterogeneity of PD-L1 and MET expression in metastatic renal cell carcinoma (mRCC). J Clin Oncol. 2017;35(15_suppl):4569.

